# Mapping Meiotic Recombination DNA Double‐Strand Breaks (DSBs) Hotspots —Methodological Advances and Challenges

**DOI:** 10.1002/ggn2.202500019

**Published:** 2026-07-14

**Authors:** Jianqiang Bao

**Affiliations:** ^1^ Key Laboratory of Reproductive Health Diseases Research and Translation NHC Key Laboratory of Tropical Disease Control Ministry of Education Hainan Medical University School of Life Sciences and Medical Technology Haikou China

**Keywords:** DSB, germline, meiosis, testis

## Abstract

Programmed DNA double‐strand breaks (DSBs) initiate meiotic recombination at discrete genomic hotspots, and their precise mapping is crucial for understanding the molecular regulation of genome stability and evolution. A variety of genome‐wide methods have been developed, each leveraging different biochemical principles — from direct capture of Spo11‐oligonucleotide covalent intermediates to sequencing protein‐bound single‐stranded DNA (ssDNA), in situ DNA end labeling, and chromatin immunoprecipitation (ChIP). Direct approaches such as Spo11‐oligo mappin, CC‐seq and in situ DNA end labeling (END‐seq) achieve nucleotide‐resolution detection of the initial cleavage site, but typically require large amounts of starting material. ChIP ssDNA‐based methods (e.g., ChIP‐SSDS) enrich for recombinase associated proteins‐ bound (DMC1, RAD51, RPA) intermediates, reflecting an indirect proxy of DSB sites, and are limited to early resection stages. Together, while these complementary methods comprise a toolbox for dissecting meiotic hotspot landscapes, a more robust and facile approach for routine detection of meiotic DSB hotspots across diverse species remains to be developed.

## 1 Introduction

**FIGURE 1:**
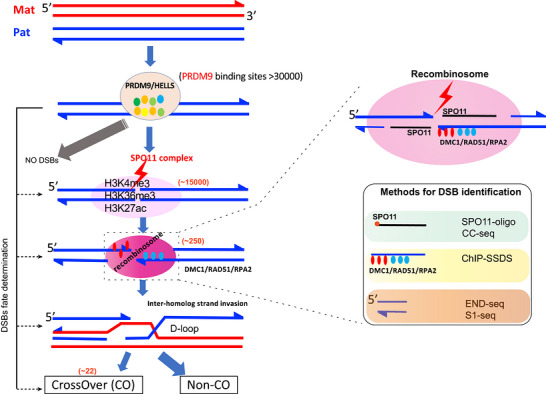
Schematic illustration summarizing the DSB generation and fate determination in meiotic spermatocytes in mammals. Left panel depicts the stepwise progression of DSB from production to repair, which ultimately commit to the crossover (CO) and Non‐CO outcome. DSB hotspots are predetermined by the binding of PRDM9 and the pioneering factor HELLS, which trigger recruitment of downstream factors for DSB processing, initiated by SPO11 cut, followed by DSB machinery repair (recombinosome). Right panel illustrates the detection approaches as designed against specific DSB substrates of DNA intermediate involved in the recombinosome machinery.

Meiosis is a specialized cell division in sexually reproducing organisms that ensures genome haploidization and genetic diversity through homologous recombination. Programmed DNA double‐strand breaks (DSBs), catalyzed by the SPO11 protein, initiate this process at specific genomic loci known as DSB hotspots [1, 2, 3]. DSB hotspots are genomic regions where DSBs occur most frequently during meiosis. Mapping these hotspots is essential for understanding recombination regulation, chromosome segregation, and their implications for reproductive health, including infertility and aneuploidies [4, 5, 6]. In most mammals, including humans and mice, the protein PRDM9 acts as the major regulator of DSB hotspot location (Figure 1). PRDM9 is a multi‐domain protein with two critical activities [7, 8, 9]. First, its C‐terminal array of C2H2 zinc fingers functions as a sequence‐specific DNA‐binding module, recognizing particular motifs in the genome. The extreme polymorphism in this zinc finger array, both between and within species, is directly responsible for the variation in hotspot locations. Second, upon binding to its target DNA sequence, PRDM9's N‐terminal PR/SET domain functions as a histone methyltransferase, depositing both H3K4 trimethylation (H3K4me3) and H3K36 trimethylation (H3K36me3) on adjacent nucleosomes (Figure 1) [4]. These histone marks laid down by PRDM9 are recognized by “reader” proteins that link the hotspot to the DSB machinery. ZCWPW1 recognizes both histone marks and interacts with axis‐associated proteins, collectively tethering the PRDM9‐bound hotspot to the chromosome axis where the DSB machinery resides [7, 10]. In organisms lacking a functional PRDM9 gene — a group that includes budding yeast (*S. cerevisiae*), plants (*Arabidopsis thaliana*), nematodes (*C. elegans*), and some vertebrates like birds and dogs — the locations of meiotic DSBs are intrinsically linked to the existing open architecture of the genome [7, 11, 12]. In these organisms, DSB hotspots are predominantly found in open, nucleosome‐depleted regions (NDRs), which are typically located at the promoter regions of protein‐coding genes and other regulatory elements. These sites are inherently accessible to the DSB machinery and are often pre‐marked with H3K4me3 as part of their normal transcriptional regulation [4, 9, 11].

The presence or absence of PRDM9 defines two divergent evolutionary paths for the initiation of meiotic recombination. The PRDM9‐independent strategy offers stability by linking recombination to conserved functional elements of the genome. In contrast, the PRDM9‐dependent strategy provides evolutionary flexibility, allowing the recombination landscape to change rapidly, but at the cost of a constant arms race between the specifying protein and its target DNA sequences [4, 9, 11]. This perspective briefly reviews the primary approaches for mapping meiotic DSB hotspots, including the recently developed CC‐seq, END‐seq and S1‐seq, evaluating their strengths, weaknesses, and applications.

## 2 Importance of DSB Hotspot Detection

DSB hotspots are critical for initiating recombination, so as to establish physical connections between homologous chromosomes to ensure proper alignment and segregation during meiosis I^5^. Errors in DSB formation or repair can lead to aneuploidies, contributing to miscarriages, infertility, and chromosomal disorders like Down syndrome [13, 14]. By promoting recombination, DSB hotspots generate new haplotype configurations in gametes, enhancing genetic diversity. This is crucial for adaptation to environmental changes and species survival. In addition, DSB hotspots influence locus‐specific rates of genetic change over evolutionary time, contributing to speciation [15, 16, 17]. For instance, studies in mice shows their role in evolutionary turnover, driving speciation. High‐resolution mapping of DSB hotspots reveals genomic and epigenetic factors governing recombination, offering insights into evolutionary biology [16], genome stability [9], and reproductive health [11, 16, 17, 18]. The methods discussed here have transformed our capability to study these processes, each providing unique perspectives on DSB localization and regulation.

## 3 Methods for Detecting DSB Hotspots

**FIGURE 2:**
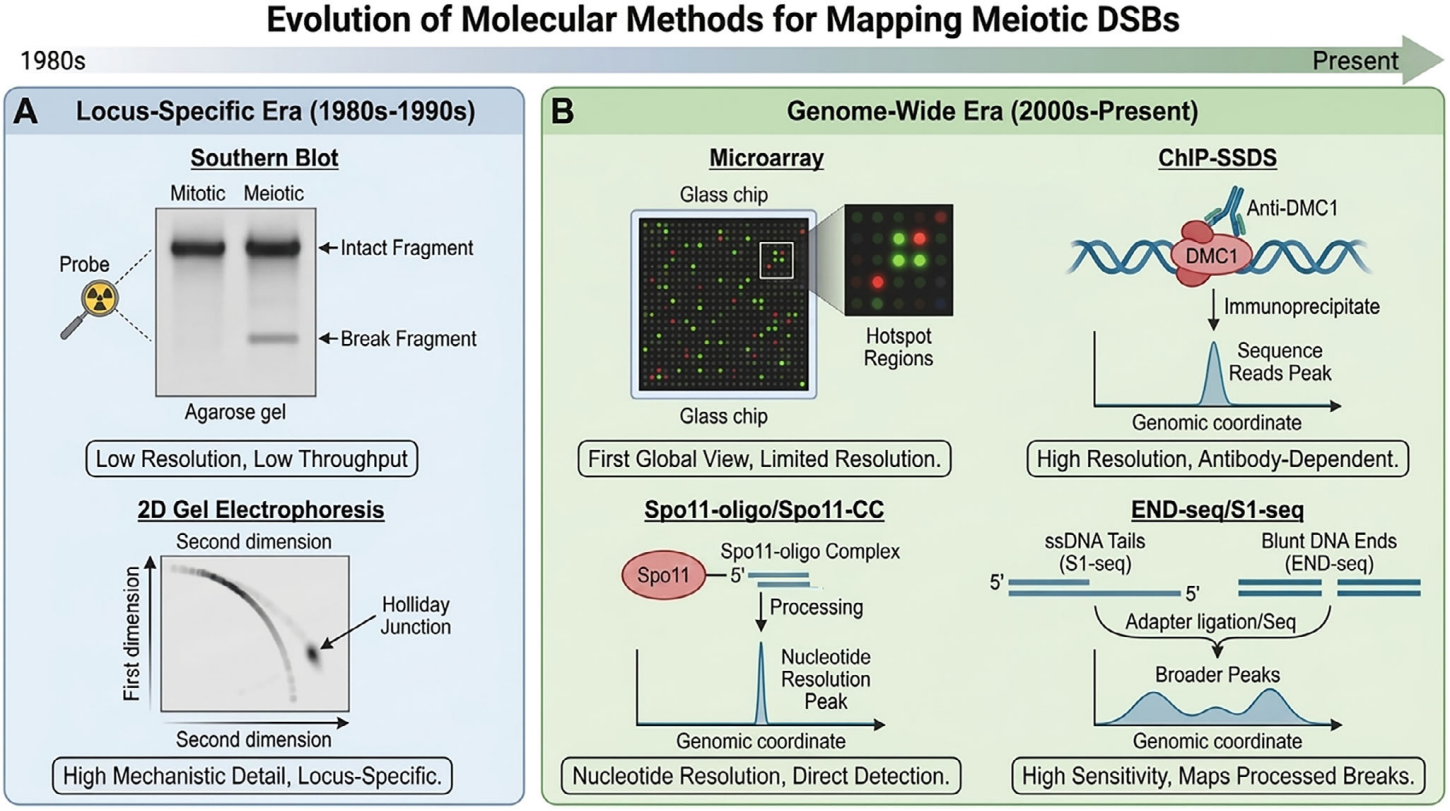
A schematic timeline illustrating the progression of key techniques for mapping meiotic DSB hotspots. (A) Locus‐Specific Era (1980s–1990s): showing Southern Blotting and 2D Gel Electrophoresis. Southern Blot indicates a restriction‐digested DNA fragment from a hotspot, with a smaller band appearing in meiotic samples, indicating a break. 2D Gel shows a stylized 2D gel with the arc of linear DNA and a distinct spot migrating off the arc. (B) Genome‐Wide Era (2000s‐Present) depicts Microarrays and various NGS‐based methods. Microarray shows a schematic of a chip with fluorescent spots indicating hotspot regions. ChIP‐SSDS shows antibodies pulling down a target protein (e.g., DMC1) bound to DNA; SPO11‐oligo indicates Spo11 covalently bound to a short oligo, which is then sequenced. END‐seq/S1‐seq show the detection of ssDNA‐dsDNA boundary, leading to broader peaks of reads.

Over the decades, a remarkable array of molecular techniques has been developed to map these initial hotspots. Prior to the era of high‐throughput sequencing genomics, identifying the precise locations of meiotic DSBs was a significant challenge. Initial approaches were often indirect or could only provide a low‐resolution view of recombination events at a handful of specific loci (Figure 2) [5, 19]. For example, Southern blotting was one of the first direct physical methods used to visualize DSBs at specific genomic regions. The method involves digesting genomic DNA with restriction enzymes, separating the fragments by size on a gel, and using a labeled DNA probe to visualize fragments from a region of interest. The appearance of smaller, “broken” DNA fragments in meiotic cells provides direct physical evidence of a DSB within that locus. It provided the first direct physical proof of meiotic DSBs at specific genomic sites; however, this technique is extremely labor‐intensive, and is limited by the location of restriction enzyme sites, typically identifying a region of several kilobases (Figure 2). Further, Two‐Dimensional (2D) Gel Electrophoresis was employed as a powerful locus‐specific technique, which allows for the visualization of branched recombination intermediates, such as Holliday junctions, which are hallmarks of active recombination (Figure 2) [20, 21, 22]. Nonetheless, these approaches are not suitable for unbiased, genome‐wide discovery of hotspots.

The development of DNA microarrays [21, 22, 23] and next‐generation sequencing (NGS) revolutionized the study of meiotic recombination, enabling genome‐wide mapping of DSB hotspots with progressively higher resolution and sensitivity (Figure 2). The following methods represent the state‐of‐the‐art approaches for detecting DSB hotspots in meiosis, each with its own advantages, disadvantages, and critical nuances, as summarized in Table 1. The choice of DSB detection method depends on experimental goals, and the cost for the time and labor: SPO11‐oligo mapping offers unparalleled resolution for initiation studies, while SSDS and END‐seq provide insights into resection and repair.

**TABLE 1:** Methods for Detecting genome‐wide DSB Hotspots in Meiosis.

Method	Description	Strengths	Weaknesses	Key Reference
Selective Enrichment of ssDNA by Benzoyl naphthoyl DEAE (BND) cellulose & microarray	BND selectively binds the DSB‐related ssDNA nucleotides. In yeast of dmc∆ mutants, they found the accumulated DSBs are more uniformly distributed even in DSB‐cold regions than was previously thought	‐most earlier and effective method in probing genome‐wide DSB locations combined with microarray	‐ limited spatial and quantitative resolution; ‐low efficiency; ‐low sensitivity	[23, 56]
**SPO11‐oligo mapping**	Immunoprecipitation and deep sequencing of SPO11‐bound single‐strand oligonucleotides to directly map DSB sites.	‐ High resolution (nucleotide level) ‐ Direct and quantitative mapping of DSB sites;—Broadly applicable to any species	‐ Requires specific antibodies ‐ May miss quickly repaired DSBs ‐ Technically challenging with high input samples and single‐strand library preparation	[21, 24, 48]
**ChIP followed by Single‐Stranded DNA Sequencing (ChIP‐SSDS)**	ChIP‐seq of ssDNA bound by DMC1 or RAD51 to map DSB‐associated ssDNA.	‐ High resolution and strand‐specific ‐ Captures ssDNA intermediates ‐ Suitable for mammals	‐ Estimate affected by ssDNA lifetime and protein loading ‐ Requires antibody optimization	[57]
				
				
**END‐seq**	Direct ligation of sequencing adapters to DSB ends for high‐resolution, unbiased genome‐wide mapping of DSBs.	‐ Unbiased mapping of DSBs ‐ informative on end resection ‐ Applicable to various species	‐Require high‐quality nuclei preparation ‐ Needs embedding in agarose plugs	[34]
**S1‐seq**	Sequencing of ssDNA‐to‐dsDNA junctions to study DNA end resection at known DSB sites during meiotic recombination.	‐ Provides base‐pair precision of end resection endpoints; ‐ ‐compatible with yeast and mammalian systems. ‐can detect unresected DSBs	‐Requires careful nuclease titration; ‐may cleave secondary structures;	[37, 45]
**CC‐seq**	Enrichment of SPO11cc oligos, followed by strand‐specific adaptor ligation and sequencing at nucleotide resolution	‐No agarose embedding;—Provides base‐pair resolution of the DSB position. ‐Antibody independent applicable in various species	‐ Not (necessarily) specific for SPO11	[39]
**DEtail‐seq**	Direct ligation of adapters to DSB 3’end to capture the 3’ ssDNA	‐ Direct and ultra‐efficiently characterizing the 3′ ends of meiotic DSBs with near single‐nucleotide resolution among various species	‐ Needs embedding in agarose plugs; ‐needs commercial kit (IDT adaptase Inc)	[33]

### 3.1 SPO11‐Oligo Mapping

SPO11‐oligo mapping involves immunoprecipitating SPO11 protein, covalently bound to short oligonucleotides during DSB formation, followed by sequencing to map break sites at nucleotide resolution (Figure 2) [24]. It has been applied in *Saccharomyces cerevisiae*, *Mus musculus*, and *Arabidopsis thaliana*. The SPO11‐oligo method has mapped 3604–4099 hotspots in yeast, 5,914 in Arabidopsis, and 13 960 in mice, revealing hotspot density and width variations [25, 26]. It is effective across species with conserved SPO11 homologs, and has the following strengths: (i) High resolution at the nucleotide level, making it a precise method for mapping the initial cleavage event; (ii) Directly detects DSB sites via SPO11‐bound DNA with a very high signal‐to‐noise ratio; (iii) Broadly applicable where SPO11 is conserved. However, this method requires specific, high‐quality antibodies against SPO11, might miss rapidly repaired DSBs or those not bound by SPO11, and is technically challenging due to purification of low‐abundance Spo11‐oligo complexes and the specialized library preparation for very short, single‐stranded oligos [27]. Further studies extended the SPO11‐oligo findings as evidenced by the “double‐cuts” or “double‐DSBs” especially prevalent in ATM‐deficient meiotic cells [28, 29].

### 3.2 Chromatin Immunoprecipitation Followed by Single‐Stranded DNA Sequencing (ChIP‐SSDS)

Once a DSB is formed, the 5' ends are resected to create long 3' single‐stranded DNA (ssDNA) tails. ChIP‐SSDS employes chromatin immunoprecipitation followed by high‐throughput sequencing (ChIP‐seq) to detect single‐stranded DNA (ssDNA) bound by strand‐exchange proteins DMC1 and RAD51 at DSB sites (Figure 2) [30]. After ChIP enrichment, a specialized library is made by exploiting the ability of ssDNA to form hairpins via microhomology. A polymerase extends the 3' end, creating a double‐stranded fragment with a unique inverted repeat that identifies it as originating from ssDNA. It is optimized for mammals, including mice and humans, as well as yeast and maize. SSDS has identified 9874–15 677 hotspots in mice and 38 946 in humans [31, 32], highlighting species‐specific recombination patterns, particularly influenced by PRDM9 in mammals. ChIP‐SSDS is effective in mammals, producing high‐resolution mapping of DSB‐associated ssDNA, and possibly captures universal ssDNA intermediates of recombination. However, ChIP‐SSDS is reliant on the kinetic enrichment step during library construction and relatively provides lower resolution for the initial cleavage event compared to SPO11‐oligo sequencing. The DSB frequency estimates vary with ssDNA intermediate lifetime and DMC1/RAD51 loading. It is less sensitive and typically demands large number of cells in organisms with low DSB frequency or heterogeneous germ cells. As a comparison, another recently developed “DEtail‐seq” technique could directly detect the exposed 3’ ssDNA end by a proprietary adaptase (from IDT Inc) in situ after the embedding of the single testicular cells, offering a straightforward way to evaluate the DSB site distribution across the host genome [33].

### 3.3 END‐Seq

END‐seq maps DSBs genome‐wide by directly ligating sequencing adapters to DSB ends after enzymatic processing (ExoVII/ExoT), providing nucleotide‐resolution 5’‐resected tract length at DSB sites (Figure 2) [34, 35, 36]. It has been optimized for human and mouse cells. While primarily used for non‐meiotic DSBs, END‐seq's unbiased approach makes it applicable to meiotic DSB hotspot mapping, particularly in mammals, where it can quantitatively map DSBs with low background and base‐pair resolution. Its key feature is the in situ processing of DNA ends within intact nuclei, which minimizes artificial breaks. Overall, END‐seq is unbiased, genome‐wide DSB mapping without prior assumptions, and provides end resection data critical for repair pathway analysis. However, it requires specific experimental conditions (e.g., agarose plugs for embeding), and is thereby labor‐intensive and time‐consuming. In addition, its sensitivity and specificity in meiotic contexts need further validation.

### 3.4 S1‐Seq

S1‐seq is a next‐generation sequencing assay that maps ssDNA‐to‐dsDNA junctions to study DNA end resection at DSB sites in both yeast and mice during meiotic recombination (Figure 2) [37, 38]. It exploits S1 nuclease to remove ssDNA tails, marking resection endpoints. It is optimized for *S. cerevisiae*, and applicable for mammalian cells. It provides high‐resolution resection data at SPO11‐induced DSBs, complementing SPO11‐oligo maps to study DSB processing dynamics. Overall, S1‐seq is applicable to meiotic recombination studies in both yeast and mammalian cells, reveals detailed molecular features of DSB processing, and exhibit high spatial resolution for end resection patterns. However, S1‐seq also necessitates cell embedding in agarose plugs, which inevitably lengthens the whole operational processing steps. Further, S1‐seq likely incurs false positive signal owing to the complexity of the secondary DNA structure as captured by S1 nuclease digestion.

### 3.5 Covalent Complex (CC)‐Seq

CC‐seq is another high‐resolution method designed to map the genome‐wide locations of the double‐stranded SPO11‐linked covalent complex (Figure 2) [39, 40, 41, 42, 43]. Its key innovation is that it is an antibody‐free, silica‐based method for enriching SPO11 covalently bound to fragmented genomic DNA, following cell lysis and DNA fragmentation. This method provides nucleotide‐resolution maps of SPO11 cleavage sites that are highly reproducible and correlate well with data from SPO11‐oligo sequencing. While optimized in *S. cerevisiae*, recent data indicate that CC‐seq was successfully used to map the DNA breaks generated by other topoisomerase‐like enzymes, such as Top2, and is readily transferable to other eukaryotic organisms [39]. However, Like SPO11‐oligo sequencing, CC‐seq requires large starting materials, and the use of nuclease‐deficient mutants (e.g., *sae2Δ, rad50S*) to accumulate sufficient covalent complexes for detection. Further, it's possible that the silica‐based enrichment is not specific to SPO11 and will possibly capture any proteins covalently bound to DNA.

## 4 Comparative Analysis

These methods listed above vary in directness, resolution, and applicability (Table 1). In general, SPO11‐oligo mapping and CC‐seq are the most direct, offering nucleotide‐level resolution [28, 39, 42, 44]. ChIP‐SSDS provides high‐resolution ssDNA mapping, but is reliant on highly specific antibody. END‐seq and S1‐seq excel in resection analysis requiring single‐cell embedding in agarose plugs and slight limitation for high‐order DNA structure variations [45, 46]. ChIP‐SSDS harnessing the ssDNA‐bound proteins is broadly adopted and valuable for regulatory studies with reduced resolution and antibody dependence. Combining these methods can provide a comprehensive view, with SPO11‐oligo mapping and SSDS defining hotspot locations, END‐seq and S1‐seq detailing DSB processing. Specifically, DMC1‐SSDS generated the first high‐resolution, genome‐wide DSB maps in mice and humans. This was a landmark achievement that was previously intractable due to the scarcity of meiotic cells and low DSB density [47]. These mammalian DSB maps were instrumental in the discovery of PRDM9 as the master regulator of hotspot location. The maps showed that hotspots were not at promoters (as in yeast) but at specific motifs that correlated with the binding sites of different PRDM9 alleles. By directly sequencing the oligonucleotides covalently bound to SPO11, SPO11‐oligo sequencing provided the first, and likely most precise, view of the exact sites of DNA cleavage at nucleotide resolution. It revealed that SPO11 does not cut DNA randomly, even within hotspots. Instead, it has a weak sequence preference, showing biases for certain nucleotides at and around the cleavage site with a degenerate 15‐bp motif [39, 44, 48]. As an antibody‐free method for capturing SPO11 covalent complexes, CC‐seq provides an essential cross‐validation of the DSB landscape and confirmed key features of SPO11 activity with high confidence [42]. Both END‐seq and S1‐seq, which maps the boundary between ssDNA and dsDNA, provided the first genome‐wide view of the extent of meiotic DSB resection [36, 45, 49]. S1‐seq can additionally map unresected meiotic DSBs with nucleotide‐resolution accuracy in Mre11‐nuclease‐deficient background [45]. It revealed that resection tracts in yeast are heterogeneous, extending for hundreds of nucleotides, and that their length is regulated by the local chromatin context. END‐seq's extremely low background allows for the highly quantitative detection of DSBs. Together, these various approaches provide us versatile and powerful tools to dissect the molecular mechanisms underlying meiotic recombination.

## 5 Perspective

These methods are complementary, enhancing our understanding of DSB hotspots. Those methods, including SPO11‐oligo mapping, S1‐seq, CC‐seq and END‐seq, could generate precise DSB maps [27, 35, 42, 45]. ChIP‐SSDS complements these by capturing ssDNA intermediates, particularly in mammals. S1‐seq provides detailed resection data, enhancing insights into DSB processing when paired with SPO11‐oligo maps [25, 37].

Emerging technologies, such as single‐cell sequencing and computational modeling, hold promise to refine DSB hotspot detection. Single‐cell approaches could address meiotic cell heterogeneity, while machine learning may predict hotspots based on genomic and epigenetic features [50, 51]. Developing accessible antibodies and standardized protocols could broaden the applicability of these approaches to non‐model organisms. Integrating these methods with functional genomics will deepen our understanding of meiotic recombination and its implications for reproductive biology.

Detecting DSB hotspots in meiosis is fundamental to molecular and reproductive biology, offering insights into genetic diversity, chromosome segregation, and genome evolution [12, 46, 48, 52, 53, 54]. The methods discussed—SPO11‐oligo mapping, ChIP‐SSDS, END‐seq, CC‐seq and S1‐seq – each contributes uniquely. The resection tract analyses deduced from both the END‐seq and S1‐seq complement established methods, enhancing our ability to study DSB dynamics. By understanding their strengths and limitations, researchers can select optimal tools, often combining approaches, to advance knowledge of meiotic recombination and its role in health and disease [53, 55]. Importantly, END‐seq and S1‐seq necessitate the embedding of testicular cells in agarose plugs, which requires lengthy time for sample processing. Therefore, a generally more quick and easy‐to‐operate protocol that can be readily set up in ordinary laboratories remains to be developed and optimized in diverse species.

## Conflicts of Interest

The authors declare no conflicts of interest.
